# Supplementary material for: Network- and systems-based re-engineering of dendritic cells with non-coding RNAs for cancer immunotherapy
Source: Theranostics. 2021 Jan 1;11(3):1412–28. doi: 10.7150/thno.53092 (PMC7738891; doi:10.7150/thno.53092)
Supplement: Supplementary file 1 — Supplementary figures. [file thnov11p1412s1.pdf]

Supplementary Materials

Network- and systems-based re-engineering of dendritic cells with non-coding RNAs for cancer immunotherapy

Xin Lai[1,5,6,*], Florian S. Dreyer[1,5,6], Martina Cantone[1,5,6], Martin Eberhardt[1,5,6], Kerstin F. Gerer[2,5,6], Tanushree Jaitly[1,5,6], Steffen Uebe[3], Christopher Lischer[1,5,6], Arif Ekici[3], Jürgen Wittmann[4], Hans-Martin Jäck[4], Niels Schaft[2,5,6], Jan Dörrie[2,5,6], Julio Vera[1,5,6,*]

1. Laboratory of Systems Tumor Immunology, Department of Dermatology, Friedrich-Alexander-Universität Erlangen-Nürnberg (FAU) and Universitätsklinikum Erlangen, Erlangen, Germany
2. RNA Group, Department of Dermatology, Friedrich-Alexander-Universität Erlangen-Nürnberg (FAU) and Universitätsklinikum Erlangen, Erlangen, Germany
3. Department of Human Genetics, Universitätsklinikum Erlangen, Erlangen, Germany
4. Division of Molecular Immunology, Department of Medicine 3, Universitätsklinikum Erlangen, Erlangen, Germany
5. Deutsches Zentrum Immuntherapie (DZI), Erlangen, Germany
6. Comprehensive Cancer Center (CCC) Erlangen, Erlangen, Germany

* To whom correspondence should be addressed. Xin Lai, Tel: +49(0)91318545888, E-mail: xin.lai@uk-erlangen.de; Julio Vera, Tel: +49(0)91318545876, E-mail: julio.vera-gonzalez@uk-erlangen.de.

Contents

SUPPLEMENTARY TEXT **2**

MicroRNA quantification 2

Reactome pathway hierarchy 2

Gene set enrichment analysis 2

Gene prioritization in regulatory networks 3

References 3

SUPPLEMENTARY FIGURES **5**

SUPPLEMENTARY TABLE CAPTIONS **14**

SUPPLEMENTARY FIGURE S2 AND S4 .... **16**

SUPPLEMENTARY TEXT

MicroRNA quantification

After using FastQC [1] to verify the quality of the samples (N and GC content, sequence quality score, and sequence duplication level), neither trimming nor adapter removal was performed. As observed in the FastQC report, a high N content is noticed towards the end of the sequences, and given the presence of the adaptor sequence at the beginning of the read, a hard-trimming procedure would have created sequences with too few remaining nucleotides, hence causing the loss of information.

As consequence, the mapping step was performed with BWA [2]. The "BWA mem" function is capable of compensating for the presence of adapters and handling short reads, thus aligning only the sequence of the read containing the miRNA. Reads were mapped to the human reference genome hg19. The obtained SAM files were split according to chromosomal location of the mapped read in order to parallelize and speed up the quantification procedure. Each chromosome-specific file was used as input for *bedtools genomecov* [3], specifying a GFF annotation file containing miRNAs only as reference. This function produces per-base counts on the reference.

To further quantify the miRNA content, a customized pipeline was designed. For characterizing the expression value for pri-miRNAs and mature miRNAs separately, the positions annotated in the miRNA-specific GFF file were extracted and then accessed in the per-base counts. The GFF file was taken from miRBase (version 21, genome id GRCh38, genome accession NCBI_Assembly:GCA_000001405.15, release 06/2014). For each interval (from start to end position of each pre- and mature-miRNA) given in the GFF file, the average count (or pseudo-count) was calculated and assigned to the annotated miRNA or pri-miRNA.

Reactome pathway hierarchy

We downloaded the Reactome pathway relation data (release 68) and created a *Homo sapiens* pathway relation network that was used to retrace pathways' hierarchy. Specifically, pathways that appear at the top of the directional network (i.e., the pathways have only outgoing edges and no ingoing edges) were regarded as root categories. Pathways belonging to a root category were identified by manual searching. We started by searching the first-neighbour terms of a root category and continued until no more first-neighbour terms could be added into the category. This resulted in a pathway category with its associated pathways. By doing so, we successfully reproduced 26 root categories from Reactome.

Gene set enrichment analysis

We applied a competitive gene set test to perform gene set enrichment analyses for Reactome pathways. The algorithm CAMERA tests whether the genes in the set are lowly or highly ranked in terms of differential expression relative to genes not in the set [4]. Specifically, genes were sorted based on their weighted log2 fold-change (i.e., log2 fold-change divided by the standard error of the log2 fold-change) from differential expression analysis to create a background gene list. The enrichment score of a gene set

was calculated by walking down the background list, increasing a local enrichment score when members of the gene set are frequently encountered and decreasing it when the associated genes are less encountered [5,6]. The maximum local enrichment score was used as an enrichment score of a gene set. A positive score means that the pathway-specific genes are more likely to be highly ranked in the gene list (i.e., their corresponding expressions tend to be upregulated in caIKK-DCs), while a negative score means that the genes are more likely to be lowly ranked (i.e., their corresponding expressions tend to be downregulated in caIKK-DCs). The corresponding p-value of a pathway was estimated after adjusting the variance of gene-wise statistics using a factor that estimates gene-wise correlation and the number of genes in the tested gene set. All obtained p-values were corrected using the Benjamini-Hochberg method. Pathways with FDR ≤ 0.05 were regarded as significantly up- (positive score) or down-regulated (negative score) in our comparison of caIKK-DCs with controls.

Gene prioritization in regulatory networks

The following formula was used to score a gene in a network

$$S_n(d) = AUC\left(\frac{2}{\overline{NW} \cdot k} \cdot \sum_m \left(NW_m - \overline{NW}\right) \boldsymbol{I}(D(n,m) < d)\right),$$

with $NW_m = -\log10(\mathrm{adjp}_m) \cdot |\log2(\mathrm{fc}_m)|$ and $\overline{NW} = \frac{1}{k}\sum_{i=1}^{k} NW_i$.

The node weights (*NW*) of a gene is calculated as the product of its absolute log2 fold change and the negative log10-transformation of the adjusted p-value, both of which were obtained through a differential gene expression analysis. $\overline{NW}$ is the average of the node weights of all network nodes. $\boldsymbol{I}(D(n,m) < d)$ is an indicator function, when the distance (*D*) between *n* and *m* is shorter than *d* it equals 1 otherwise it equals 0. The distance between two nodes was calculated and normalized using the equation *D*(*n*,*m*) = 1 – $|p|$, where *p* represents the Pearson correlation coefficient between two interacting genes. The equation assigns an interaction with strong correlation a shorter distance, indicating that the perturbation of the source gene is more likely to activate or inhibit the expression of the target gene. *d* is a network-customized, topology-dependent distance threshold between pairs of nodes, and the distance is calculated using the diffusion kernel-based method that incorporates distances along multiple paths between pairs of genes [7]. Before calculation *d* is discretized into corresponding bins. Then, *d* increases from 0 to the diameter of the network (i.e., the maximum distance between nodes) by step 1, and for each step we computed a value, resulting in a curve that always ends at 0 when *d* reaches its maximum. The resulted area under the curve (AUC) is defined as the score of a node $S_n(d)$ and the score is used to prioritize genes in a network.

References

1. Andrews S. Babraham Bioinformatics - FastQC A Quality Control tool for High Throughput Sequence Data. 2017. Available at: http://www.bioinformatics.babraham.ac.uk/projects/fastqc/
2. Li H. Aligning sequence reads, clone sequences and assembly contigs with BWA-MEM. arXiv:13033997. 2013. Available at: http://arxiv.org/abs/1303.3997
3. Quinlan AR, Hall IM. BEDTools: a flexible suite of utilities for comparing genomic features. Bioinformatics. 2010; 26: 841–2.

4. Wu D, Smyth GK. Camera: a competitive gene set test accounting for inter-gene correlation. Nucleic Acids Res. 2012; 40: e133–e133.
5. Subramanian A, Tamayo P, Mootha VK, et al. Gene set enrichment analysis: a knowledge-based approach for interpreting genome-wide expression profiles. Proc Natl Acad Sci USA. 2005; 102: 15545–50.
6. Reimand J, Isserlin R, Voisin V, et al. Pathway enrichment analysis and visualization of omics data using g:Profiler, GSEA, Cytoscape and EnrichmentMap. Nature Protocols. 2019; 14: 482–517.
7. Cornish AJ, Markowetz F. SANTA: quantifying the functional content of molecular networks. PLoS Comput Biol. 2014; 10: e1003808.

SUPPLEMENTARY FIGURES

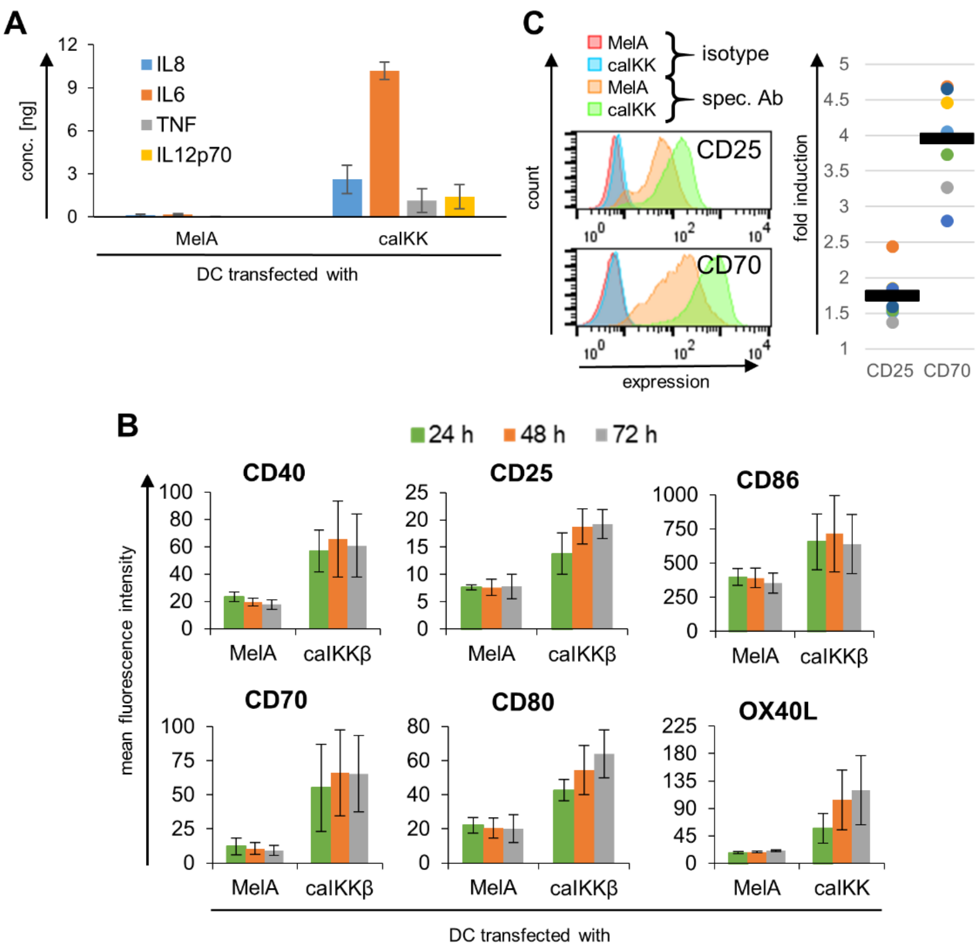

Figure S1. Efficiency of electroporation of caIKK in DCs. DC were prepared identically to those used for RNA-sequencing: cocktail-matured DCs were electroporated with mRNA, encoding constitutively active IKKß (caIKK) or, as negative control, Melan-A (MelA). (**A**) After 24 h of incubation, supernatants were harvested and the concentrations of the indicated inflammatory cytokines were determined. The average ± standard deviation of three independent experiments is shown. (**B**) Electroporated DCs were harvested 24 h, 48 h, and 72 h after electroporation, and the expression of the indicated surface markers was determined. Isotype background values were subtracted. The average ± standard deviation of three independent experiments is shown. (**C**) From all DC-preparations, which were used for RNA sequencing, an aliquot was cultured for 24 h after electroporation. To confirm activation of NF-κB, CD25 and CD70 expression were measured. The left panel shows a representative histogram, and the right panel depicts data from the seven donors used in the study. The fold-induction was calculated by dividing the mean fluorescence intensity of the caIKK-transfected cells by that of the MelA-transfected ones. The black bar indicates the average.

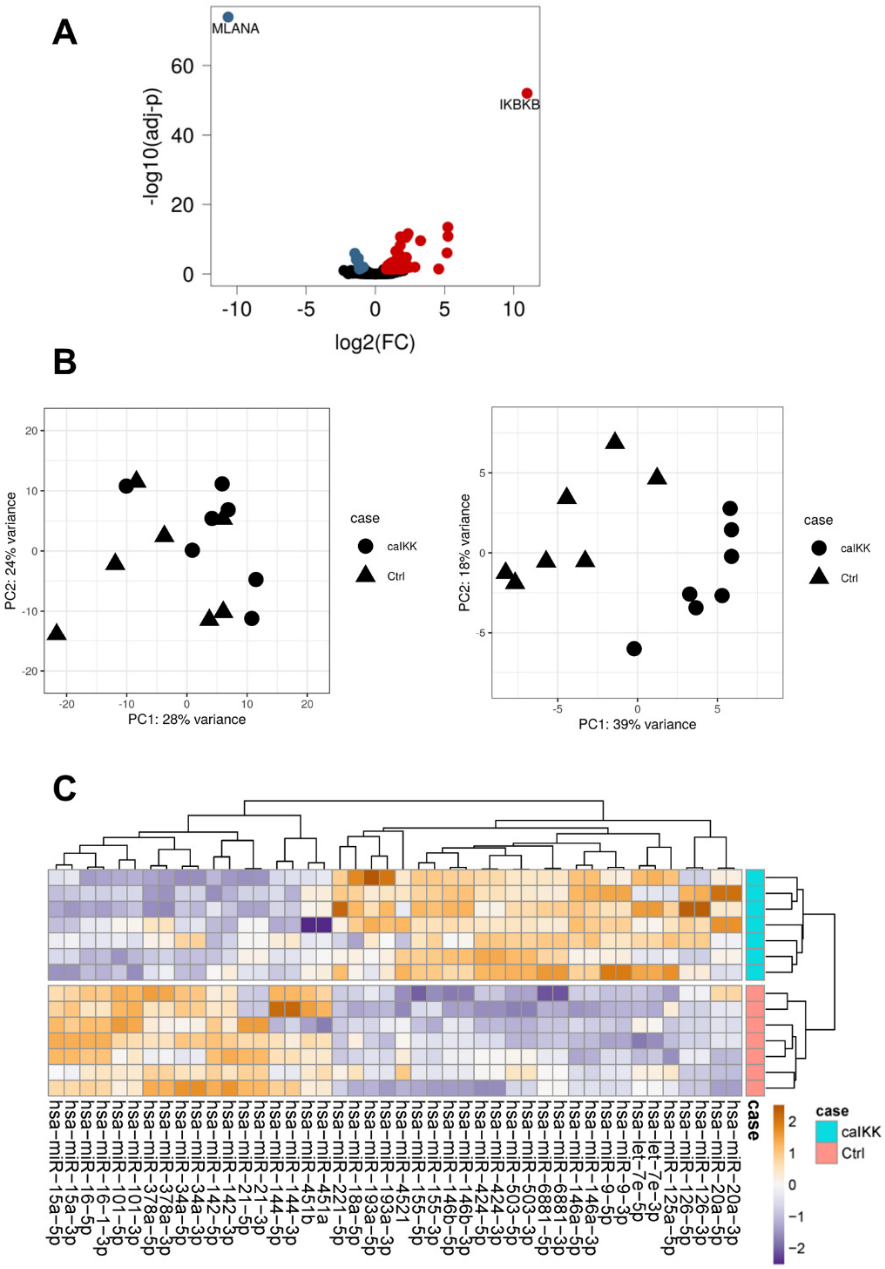

Figure S2. Summary of a differential gene expression analysis for the complete transcriptome in caIKK-DCs (caIKK) and controls (Ctrl) obtained from seven donors. (**A**) The volcano plot shows significantly (FDR ≤ 0.05) up- (red) and down-regulated (blue) genes in caIKK-DCs. *MLANA* encoding Melan-A and *IKBKB* encoding IKKβ are identified as the most differentially expressed genes. (**B**) The PCA plot shows the distribution of DC samples in a principal component analysis using the complete transcriptome data (left) or only the miRNA expression data (right) as input. (**C**) The heat map shows a hierarchical clustering of the DC samples using the 44 differentially expressed miRNAs. The grid cells show the miRNA-wise z-score of expression. The hierarchical clustering was performed using Euclidean distance with the average linkage algorithm.

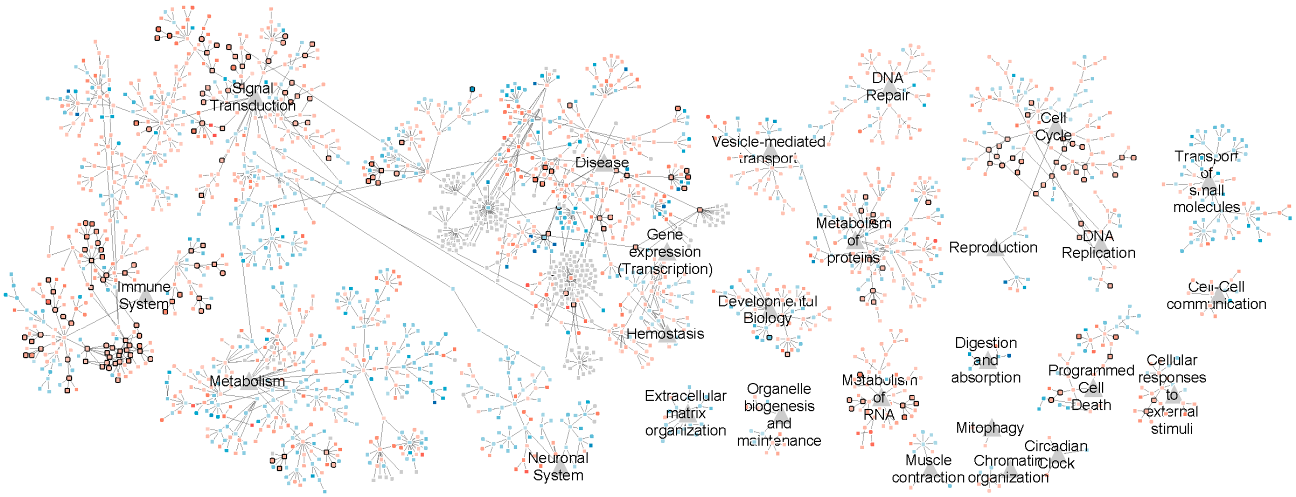

Figure S3. Overview of pathways in which the differentially expressed genes are enriched. Pathways from Reactome organized according to its pathway categories (labelled grey triangles) are shown as square nodes. The corresponding enrichment score is shown as the node's fill colour (red: positive score; blue: negative score). Square grey nodes represent pathways that have no enrichment score because they do not contain genes but other molecules, such as chemical compounds and small molecules. Nodes with black borders are identified as significantly enriched pathways (FDR ≤ 0.05). The network was arranged using prefuse force-directed layout before manual adaptation. A support vector format of the figure can be found in the separated PDF file.

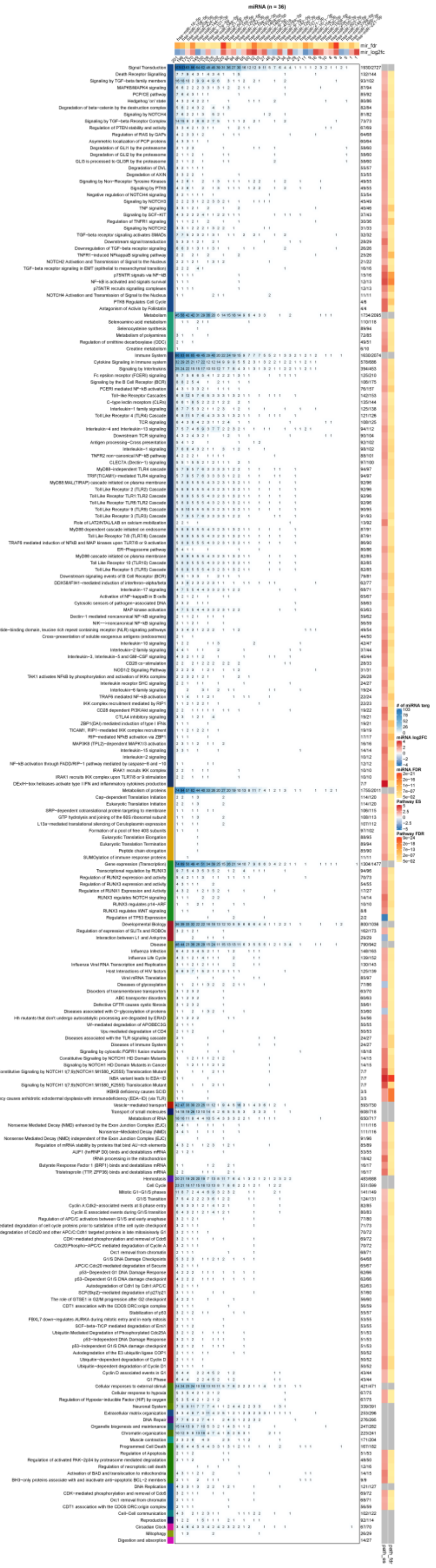

Figure S4. miRNA targeting profiles in enriched Reactome pathways. The heat map shows the number of protein-coding genes targeted by the DE miRNAs in the significantly enriched pathways (FDR ≤ 0.05). The pathways are clustered by the 26 primary Reactome categories (colour code on the left side). Each cluster begins with a category term followed by the enriched pathways belonging to this category.

Some pathways are associated with multiple categories, e.g., *synthesis of DNA* and *DNA replication pre-initiation* are can be found in *cell cycle* and *DNA replication*. The top annotation shows statistics of differential expression analysis of the miRNAs (i.e., log2 fold-change and FDR) and the number of the terms (including pathways and categories) regulated by the miRNA. The right annotation show the results of the gene set enrichment analysis (enrichment scores and FDR), excluding the 26 category terms (grey). On the right side, the number of genes that were found in our RNA-seq data and the total number of molecules of a category or a pathway are given. A support vector format of the figure can be found in the separated PDF file.

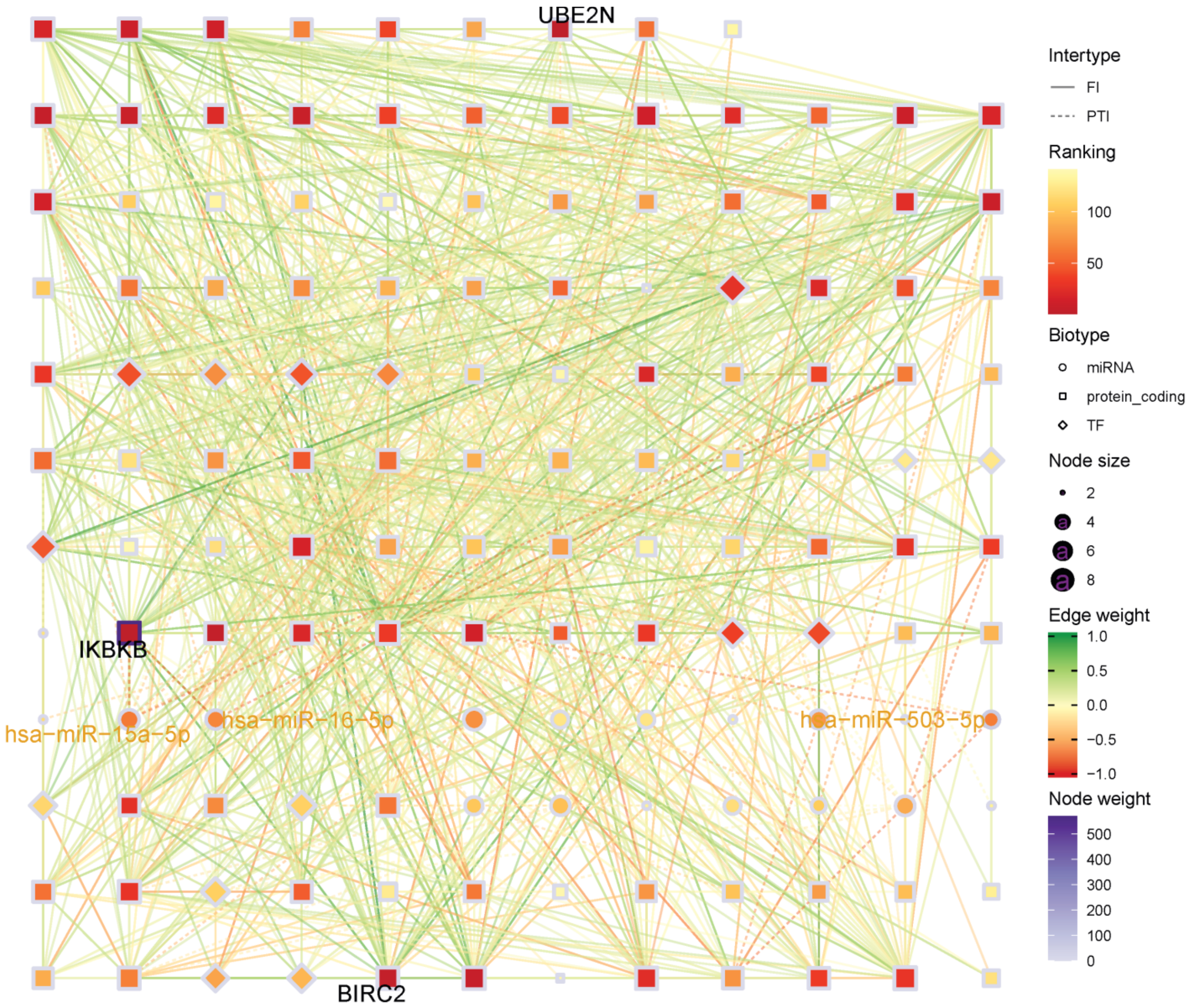

Figure S5. Illustration of gene prioritization results. The gene prioritization method was used to rank genes in a regulatory network that underlies the *Toll-like Receptor 4 Cascade* (R-HSA-166016) from the Reactome category *immune system*. The network contains three types of molecular species (i.e., miRNAs, protein-coding genes, and TFs) and two types of molecular interactions such as functional interactions (FI) among protein-coding genes and post-transcriptional interactions (PTI) mediated by miRNAs. The size of a node is proportional to its node degree (i.e., the number of edges connected to the node). The colour of its border denotes a node's expression perturbation. Node colour represents their ranking in the network (red is the highest ranking and yellow is the lowest). Edge colour indicates Pearson correlation coefficients between genes. The top three ranking miRNAs and genes are labelled.

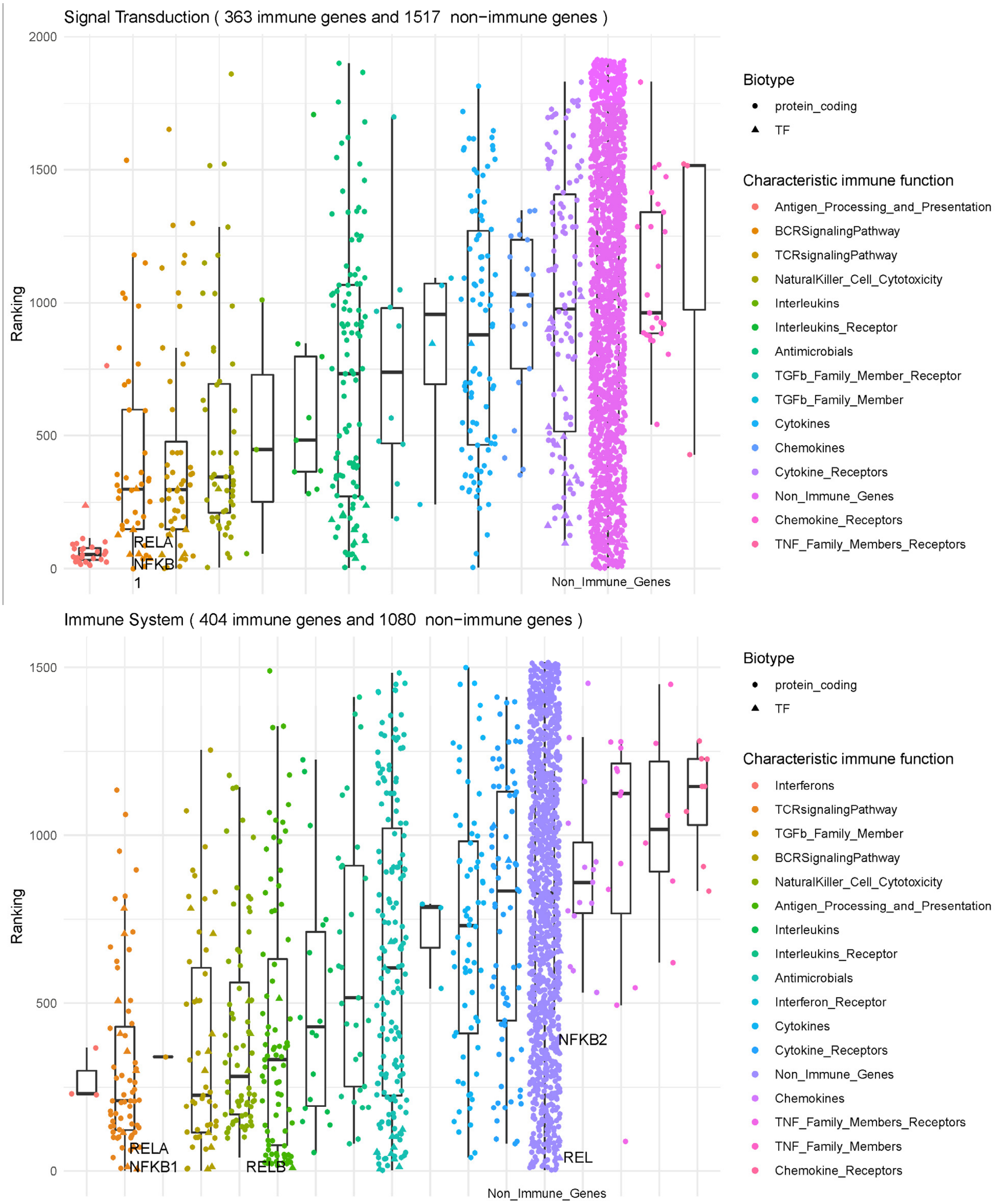

Figure S6. Network prioritisation-derived ranks of protein-coding genes broken down according to their characteristic immune function. The box plots show the ranks of all protein-coding genes (points) appearing in the reconstructed regulatory networks from the *signal transduction* (top) and *immune system* (bottom) pathway categories, respectively. TFs are drawn in triangles and NF-κB family members (i.e., *NFKB1*, *NFKB2*, *RELA*, *RELB*, and *REL*) are labelled with their names. Genes with smaller values rank

higher in the networks. Genes are classified according to their characteristic function in the immune system, with the non-immune genes collected in a separate group that is overlaid by the highest number of genes. The boxes are ordered based on the average ranking of the involved genes, beginning with the highest average ranking on the left.

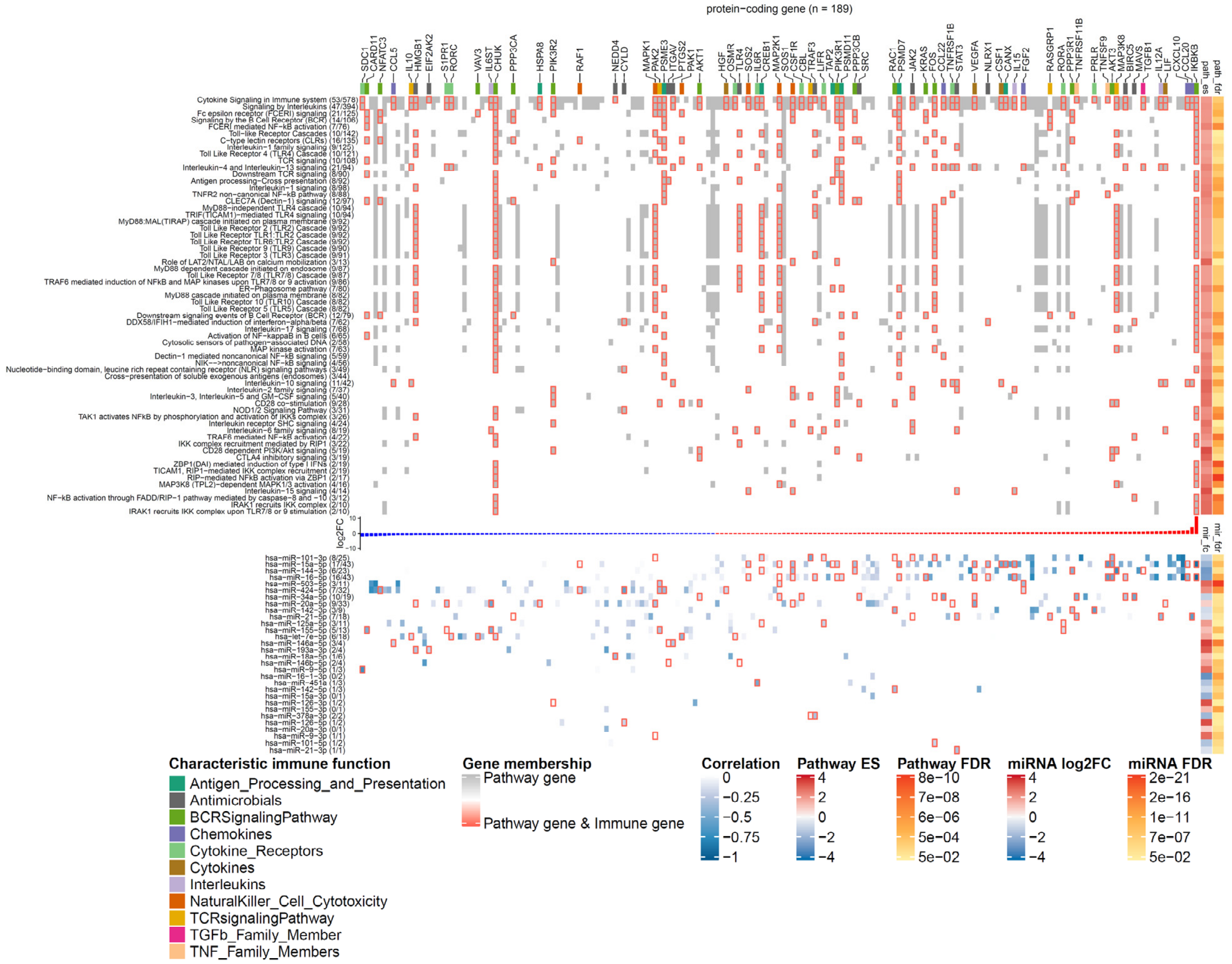

Figure S7. miRNA-gene interactions in immune signalling pathways. Identified miRNA-mediated gene regulation in pathways associated with the category *immune system*. This heat map shows all significantly enriched pathways in the category and all identified miRNA-gene interactions (Pearson correlation < 0). The annotation of the heat map is the same as in Figure 5 in the main text.

SUPPLEMENTARY TABLE CAPTIONS

Supplementary Table S1 Read counts of the annotated protein-coding genes. The mapped and annotated read counts of protein-coding genes in DCs. The DC samples were collected from 7 donors (S1-S7) and divided into two groups (caIKK-DC and Ctrl).

Supplementary Table S2 Read counts of the annotated miRNAs. The mapped and annotated read counts of miRNAs in DCs. The DC samples were collected from 7 donors (S1-S7) and divided into two groups (caIKK-DC and Ctrl).

Supplementary Table S3 Results of differential gene expression analysis of protein-coding genes. The table columns are Ensembl gene identifier, gene symbol, base mean of gene expression in all DC samples, log2 fold-change (caIKK-DC vs Ctrl), calculated statistics of Wald test, p-value, and adjusted p-value calculated using the Benjamini Hochberg method. Genes whose adjusted p-values are NA are those filtered out by independent filtering for multiple comparisons. The rows highlighted in yellow are the identified significantly differentially expressed protein-coding genes.

Supplementary Table S4 Results of differential gene expression analysis of miRNAs. The table columns are miRbase accession identifier, miRNA name, base mean of miRNA expression in all DC samples, log2 fold-change (caIKK-DC vs Ctrl), calculated statistics of Wald test, p-value, and adjusted p-value calculated using the Benjamini Hochberg method. miRNAs whose adjusted p-values are NA are those filtered out by independent filtering for multiple comparisons. The rows highlighted in yellow are the identified significantly differentially expressed miRNAs.

Supplementary Table S5 Results of gene set enrichment analysis. The table contains the results of a gene set enrichment analysis using Reactome pathways as gene sets. The table columns are the description of pathways, the Reactome identifiers, the category of a pathway, total number of molecules of a pathway, the number of identified genes from our RNA-seq data in a pathway, calculated enrichment score of a pathway, p-value and FDR of the enrichment analysis, and list of genes appearing in the pathway. A pathway may appear several times when it is associated with several categories. Category terms are highlighted in yellow and pathways associated with NF-κB signalling are highlighted in purple.

Supplementary Table S6 Curated miRNA-gene interactions. The table contains identified miRNA-gene interactions. The green columns are the general information about miRNA and their targeting genes including miRNA-gene interaction identifier, official gene symbol, transcript identifier of target genes, miRbase identifier of miRNAs, miRNA names, and miRNA family. The purple columns contain predictions about miRNA-gene interactions by TargetScan, including seed sequence of miRNA, mature sequences of miRNAs, total number of conserved/non-conserved miRNA binding sites as well as the number of different types of binding sites such as 8mer, 7mer_m8, 7mer_1a and 6mer, and cumulative weighted context scores for the putative miRNA binding sites. The cyan columns are experimental validation of miRNA-gene interactions from miRTarbase including the identifier of the database, types of experiments used to

validate the interactions (i.e., strong and weak evidence that denotes low and high throughput experimental validation, respectively), and the corresponding PubMed identifiers of the corresponding studies. The orange columns are information from starBase including the putative miRNA binding sites supported by the number of Ago CLIP-seq experiments that shows intersection of the miRNA binding sites with binding sites of Ago protein. The red column is the calculated Pearson correlation coefficients between miRNAs and their targeted genes using our RNA-seq data.

Supplementary Table S7 Ranking of *IKBKB* in pathways. The table contains the ranking of *IKBKB* in enriched pathways. The columns are gene names, ranking of genes, Reactome pathway name, Reactome identifier, category of pathways, and the results of gene set enrichment analysis.

Supplementary Table S8 Immune genes related with DC-mediated immune response. The table contains a list of immune genes that are related with immunological function of DCs. The columns are official gene symbols, characteristic immune function, and their expression profiles including base mean, log2 fold-change, p-value, and adjusted p-value. The genes mentioned in the main text are highlighted in yellow. NA: not available.

Supplementary Table S9 Identified miRNA-gene interactions for the pathway category *immune system*. The table contains a list of identified miRNA-gene interactions for the pathways under the category of *immune system*. The columns are miRNA names, official gene symbols of target genes, and Pearson correlation coefficients.

Supplementary Table S10 Association between gene and DC phenotypes. The table contains annotated gene-phenotype associations curated according to a literature review. The columns are categories of genes, official gene symbols, regulation of DC phenotypes by genes, DC phenotypes, and corresponding references.

Figure S2

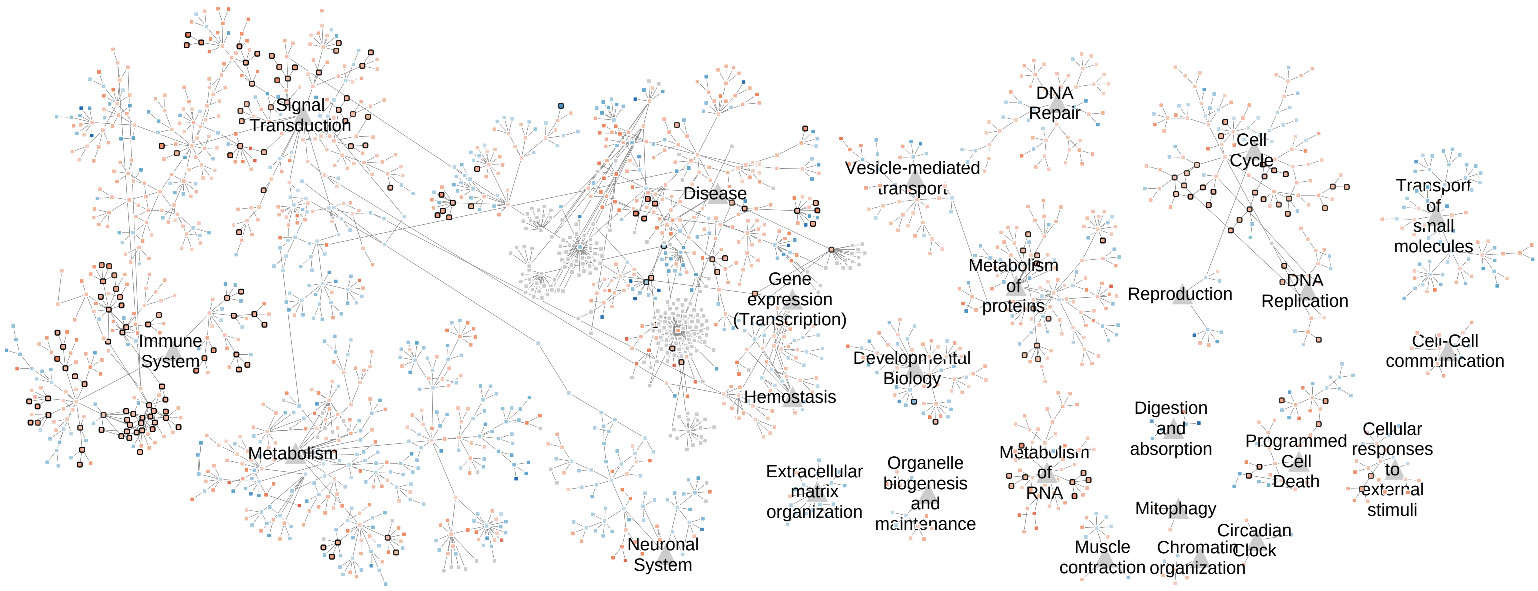

Signal Transduction
Immune System
Metabolism
Disease
Gene expression (Transcription)
Hemostasis
Neuronal System
Vesicle-mediated transport
DNA Repair
Metabolism of proteins
Developmental Biology
Extracellular matrix organization
Organelle biogenesis and maintenance
Metabolism of RNA
Cell Cycle
Reproduction
DNA Replication
Transport of small molecules
Cell-Cell communication
Digestion and absorption
Programmed Cell Death
Cellular responses to external stimuli
Mitophagy
Circadian Clock
Muscle contraction
Chromatin organization

Figure S4

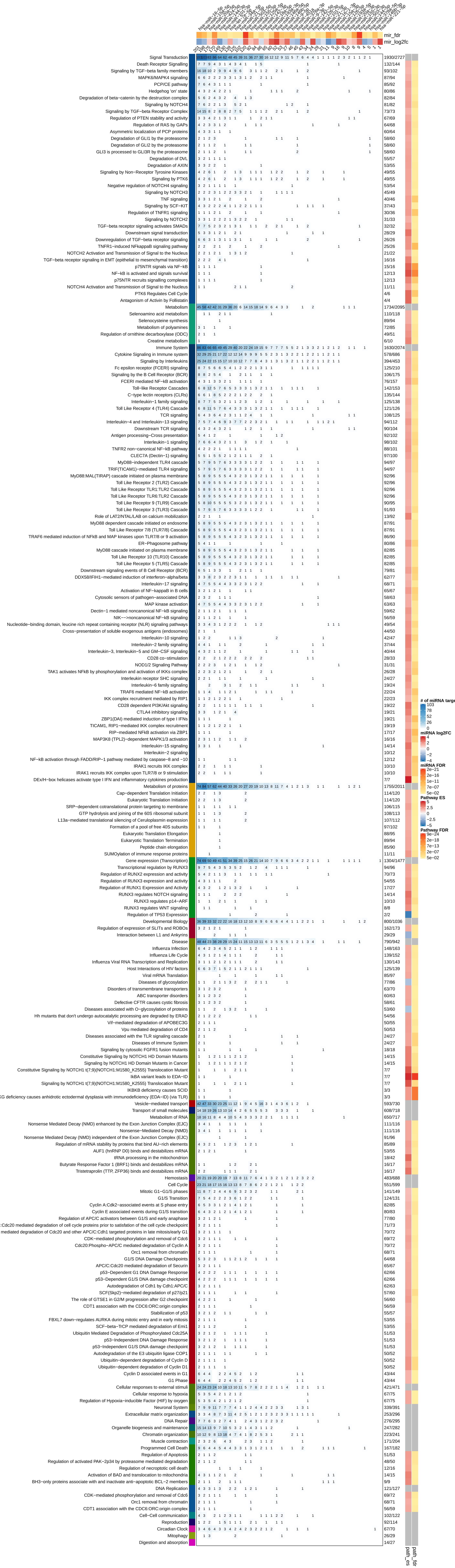

miRNA (n = 36)
mir_fdr
mir_log2fc
# of miRNA targets
103
78
52
26
0
miRNA log2FC
4
2
0
−2
−4
miRNA FDR
2e−21
2e−16
1e−11
7e−07
5e−02
Pathway ES
5
2.5
0
−2.5
−5
Pathway FDR
9e−24
2e−18
7e−13
2e−07
5e−02
path_fdr
path_es
Signal Transduction
Death Receptor Signalling
Signaling by TGF−beta family members
MAPK6/MAPK4 signaling
PCP/CE pathway
Hedgehog 'on' state
Degradation of beta−catenin by the destruction complex
Signaling by NOTCH4
Signaling by TGF−beta Receptor Complex
Regulation of PTEN stability and activity
Regulation of RAS by GAPs
Asymmetric localization of PCP proteins
Degradation of GLI1 by the proteasome
Degradation of GLI2 by the proteasome
GLI3 is processed to GLI3R by the proteasome
Degradation of DVL
Degradation of AXIN
Signaling by Non−Receptor Tyrosine Kinases
Signaling by PTK6
Negative regulation of NOTCH4 signaling
Signaling by NOTCH3
TNF signaling
Signaling by SCF−KIT
Regulation of TNFR1 signaling
Signaling by NOTCH2
TGF−beta receptor signaling activates SMADs
Downstream signal transduction
Downregulation of TGF−beta receptor signaling
TNFR1−induced NFkappaB signaling pathway
NOTCH2 Activation and Transmission of Signal to the Nucleus
TGF−beta receptor signaling in EMT (epithelial to mesenchymal transition)
p75NTR signals via NF−kB
NF−kB is activated and signals survival
p75NTR recruits signalling complexes
NOTCH4 Activation and Transmission of Signal to the Nucleus
PTK6 Regulates Cell Cycle
Antagonism of Activin by Follistatin
Metabolism
Selenoamino acid metabolism
Selenocysteine synthesis
Metabolism of polyamines
Regulation of ornithine decarboxylase (ODC)
Creatine metabolism
Immune System
Cytokine Signaling in Immune system
Signaling by Interleukins
Fc epsilon receptor (FCERI) signaling
Signaling by the B Cell Receptor (BCR)
FCERI mediated NF−kB activation
Toll−like Receptor Cascades
C−type lectin receptors (CLRs)
Interleukin−1 family signaling
Toll Like Receptor 4 (TLR4) Cascade
TCR signaling
Interleukin−4 and Interleukin−13 signaling
Downstream TCR signaling
Antigen processing−Cross presentation
Interleukin−1 signaling
TNFR2 non−canonical NF−kB pathway
CLEC7A (Dectin−1) signaling
MyD88−independent TLR4 cascade
TRIF(TICAM1)−mediated TLR4 signaling
MyD88:MAL(TIRAP) cascade initiated on plasma membrane
Toll Like Receptor 2 (TLR2) Cascade
Toll Like Receptor TLR1:TLR2 Cascade
Toll Like Receptor TLR6:TLR2 Cascade
Toll Like Receptor 9 (TLR9) Cascade
Toll Like Receptor 3 (TLR3) Cascade
Role of LAT2/NTAL/LAB on calcium mobilization
MyD88 dependent cascade initiated on endosome
Toll Like Receptor 7/8 (TLR7/8) Cascade
TRAF6 mediated induction of NFkB and MAP kinases upon TLR7/8 or 9 activation
ER−Phagosome pathway
MyD88 cascade initiated on plasma membrane
Toll Like Receptor 10 (TLR10) Cascade
Toll Like Receptor 5 (TLR5) Cascade
Downstream signaling events of B Cell Receptor (BCR)
DDX58/IFIH1−mediated induction of interferon−alpha/beta
Interleukin−17 signaling
Activation of NF−kappaB in B cells
Cytosolic sensors of pathogen−associated DNA
MAP kinase activation
Dectin−1 mediated noncanonical NF−kB signaling
NIK−−>noncanonical NF−kB signaling
Nucleotide−binding domain, leucine rich repeat containing receptor (NLR) signaling pathways
Cross−presentation of soluble exogenous antigens (endosomes)
Interleukin−10 signaling
Interleukin−2 family signaling
Interleukin−3, Interleukin−5 and GM−CSF signaling
CD28 co−stimulation
NOD1/2 Signaling Pathway
TAK1 activates NFkB by phosphorylation and activation of IKKs complex
Interleukin receptor SHC signaling
Interleukin−6 family signaling
TRAF6 mediated NF−kB activation
IKK complex recruitment mediated by RIP1
CD28 dependent PI3K/Akt signaling
CTLA4 inhibitory signaling
ZBP1(DAI) mediated induction of type I IFNs
TICAM1, RIP1−mediated IKK complex recruitment
RIP−mediated NFkB activation via ZBP1
MAP3K8 (TPL2)−dependent MAPK1/3 activation
Interleukin−15 signaling
Interleukin−2 signaling
NF−kB activation through FADD/RIP−1 pathway mediated by caspase−8 and −10
IRAK1 recruits IKK complex
IRAK1 recruits IKK complex upon TLR7/8 or 9 stimulation
DEx/H−box helicases activate type I IFN and inflammatory cytokines production
Metabolism of proteins
Cap−dependent Translation Initiation
Eukaryotic Translation Initiation
SRP−dependent cotranslational protein targeting to membrane
GTP hydrolysis and joining of the 60S ribosomal subunit
L13a−mediated translational silencing of Ceruloplasmin expression
Formation of a pool of free 40S subunits
Eukaryotic Translation Elongation
Eukaryotic Translation Termination
Peptide chain elongation
SUMOylation of immune response proteins
Gene expression (Transcription)
Transcriptional regulation by RUNX3
Regulation of RUNX2 expression and activity
Regulation of RUNX3 expression and activity
Regulation of RUNX1 Expression and Activity
RUNX3 regulates NOTCH signaling
RUNX3 regulates p14−ARF
RUNX3 regulates WNT signaling
Regulation of TP53 Expression
Developmental Biology
Regulation of expression of SLITs and ROBOs
Interaction between L1 and Ankyrins
Disease
Influenza Infection
Influenza Life Cycle
Influenza Viral RNA Transcription and Replication
Host Interactions of HIV factors
Viral mRNA Translation
Diseases of glycosylation
Disorders of transmembrane transporters
ABC transporter disorders
Defective CFTR causes cystic fibrosis
Diseases associated with O−glycosylation of proteins
Hh mutants that don't undergo autocatalytic processing are degraded by ERAD
Vif−mediated degradation of APOBEC3G
Vpu mediated degradation of CD4
Diseases associated with the TLR signaling cascade
Diseases of Immune System
Signaling by cytosolic FGFR1 fusion mutants
Constitutive Signaling by NOTCH1 HD Domain Mutants
Signaling by NOTCH1 HD Domain Mutants in Cancer
Constitutive Signaling by NOTCH1 t(7;9)(NOTCH1:M1580_K2555) Translocation Mutant
IkBA variant leads to EDA−ID
Signaling by NOTCH1 t(7;9)(NOTCH1:M1580_K2555) Translocation Mutant
IKBKB deficiency causes SCID
IKBKG deficiency causes anhidrotic ectodermal dysplasia with immunodeficiency (EDA−ID) (via TLR)
Vesicle−mediated transport
Transport of small molecules
Metabolism of RNA
Nonsense Mediated Decay (NMD) enhanced by the Exon Junction Complex (EJC)
Nonsense−Mediated Decay (NMD)
Nonsense Mediated Decay (NMD) independent of the Exon Junction Complex (EJC)
Regulation of mRNA stability by proteins that bind AU−rich elements
AUF1 (hnRNP D0) binds and destabilizes mRNA
tRNA processing in the mitochondrion
Butyrate Response Factor 1 (BRF1) binds and destabilizes mRNA
Tristetraprolin (TTP, ZFP36) binds and destabilizes mRNA
Hemostasis
Cell Cycle
Mitotic G1−G1/S phases
G1/S Transition
Cyclin A:Cdk2−associated events at S phase entry
Cyclin E associated events during G1/S transition
Regulation of APC/C activators between G1/S and early anaphase
APC:Cdc20 mediated degradation of cell cycle proteins prior to satisfation of the cell cycle checkpoint
APC/C:Cdh1 mediated degradation of Cdc20 and other APC/C:Cdh1 targeted proteins in late mitosis/early G1
CDK−mediated phosphorylation and removal of Cdc6
Cdc20:Phospho−APC/C mediated degradation of Cyclin A
Orc1 removal from chromatin
G1/S DNA Damage Checkpoints
APC/C:Cdc20 mediated degradation of Securin
p53−Dependent G1 DNA Damage Response
p53−Dependent G1/S DNA damage checkpoint
Autodegradation of Cdh1 by Cdh1:APC/C
SCF(Skp2)−mediated degradation of p27/p21
The role of GTSE1 in G2/M progression after G2 checkpoint
CDT1 association with the CDC6:ORC:origin complex
Stabilization of p53
FBXL7 down−regulates AURKA during mitotic entry and in early mitosis
SCF−beta−TrCP mediated degradation of Emi1
Ubiquitin Mediated Degradation of Phosphorylated Cdc25A
p53−Independent DNA Damage Response
p53−Independent G1/S DNA damage checkpoint
Autodegradation of the E3 ubiquitin ligase COP1
Ubiquitin−dependent degradation of Cyclin D
Ubiquitin−dependent degradation of Cyclin D1
Cyclin D associated events in G1
G1 Phase
Cellular responses to external stimuli
Cellular response to hypoxia
Regulation of Hypoxia−inducible Factor (HIF) by oxygen
Neuronal System
Extracellular matrix organization
DNA Repair
Organelle biogenesis and maintenance
Chromatin organization
Muscle contraction
Programmed Cell Death
Regulation of Apoptosis
Regulation of activated PAK−2p34 by proteasome mediated degradation
Regulation of necroptotic cell death
Activation of BAD and translocation to mitochondria
BH3−only proteins associate with and inactivate anti−apoptotic BCL−2 members
DNA Replication
CDK−mediated phosphorylation and removal of Cdc6
Orc1 removal from chromatin
CDT1 association with the CDC6:ORC:origin complex
Cell−Cell communication
Reproduction
Circadian Clock
Mitophagy
Digestion and absorption
